# Recovery of the Ratio of Closure Time during Blink Time in Lacrimal Passage Intubation

**DOI:** 10.3390/jcm12113631

**Published:** 2023-05-23

**Authors:** Yuri Kim, Helen Lew

**Affiliations:** Department of Ophthalmology, Bundang CHA Medical Center, CHA University, Seongnam 13496, Republic of Korea; a206014@chamc.co.kr

**Keywords:** blink index, ocular surface interferometer, nasolacrimal duct obstruction, closure time

## Abstract

(1) Background: We aim to find a novel blink parameter in nasolacrimal duct obstruction (NDO) patients and analyze parameters that could reflect subjective symptoms and objective indicators at the same time through a blink dynamic analysis. (2) Methods A retrospective study was conducted with 34 patients (48 eyes) who underwent lacrimal passage intubation (LPI) and 24 control groups (48 eyes). All patients’ blink patterns were measured using an ocular surface interferometer before and after LPI, including total blink (TB) and partial blink (PB) and the blink indices blink time (BT), lid closing time (LCT), closure time (CT), lid opening time (LOT), interblink time (IBT), closing speed (CS) and opening speed (OS). The tear meniscus height (TMH) was measured, and the questionnaire “Epiphora Patient’s Quality of Life (E-QOL),” which includes daily activity restriction as well as static and dynamic activities, was completed. (3) Results: Compared to CT and the ratio of CT during BT (CT/BT) in control (89.4 ± 20.0 msec, 13.16%), those in NDOs were longer (140.3 ± 92.0 msec, 20.20%) and were also related to TMH. After LPI, CT and CT/BT were recovered to 85.4 ± 22.07 msec, 13.29% (*p* < 0.001). CT and CT/BT showed a positive correlation with the E-QOL questionnaire score, particularly with dynamic activities. (4) Conclusions: CT and CT/BT, which are objective indicators associated with subjective symptoms of patients, are considered new blink indices for the evaluation of NDO patients with Munk’s score.

## 1. Introduction

There are two types of causes for epiphora: primary and secondary. Sung et al. developed the punctal reserve (PR) as a novel and clinically beneficial index for evaluating punctum parameters in nasolacrimal duct obstruction (NDO) patients using spectralis anterior segment optical coherence tomography (AS-OCT) scans [1].

There are various subjective ways for measuring the severity of epiphora, including Munk’s score, the watery eye quality of life (WEQOL) questionnaire, and the TEARS (Times wiping eyes, Effects, Activities affected, Reflex tearing, Success) score [2,3]. The widely renowned Munk’s score ranges from 0 to 5 and depicts the subjective discomfort of patients; however, there is a limit to just checking the daily numbers of wiping. To circumvent this constraint, the WEQOL questionnaire has been developed to assess the quality of life of epiphora patients [3]. The TEARS score gives a short and simple summary of the subjective and objective clinical severity of epiphora in patients, which can be utilized in a busy clinical setting [2]. TMH and PR are used to perform an objective evaluation of the amount of collected tears. Depending on the patient, the TMH and PR may objectively indicate the amount of collected tears, but further studies are necessary to investigate the subjective experiences of discomfort.

It is difficult to immediately detect subjective symptoms and objective indicators of tears due to the complexity of epiphora. Indicators that may simultaneously assess the subjective symptoms of patients and the objective parameters of epiphora are required for more precise diagnosis and evaluation.

NDO patients have a peculiar blinking pattern in which they squeeze their eyelids, and we attempted to identify novel indications by analyzing their blinking patterns. When NDO patients close their eyelids, they typically squeeze them as well. Thus, the pattern of their blinking is peculiar. By analyzing the blink pattern, we aimed to determine if it could be an additional diagnostic method of NDO.

A blink is an accurate reflection of the ocular surface and is related to the tear film’s stability and the health of the ocular surface [4]. During a 20 s blink imaging analysis, blink patterns and blink dynamic indices were identified in blepharospasm, a facial nerve palsy [5,6]. In this study, we analyzed the link dynamic index for NDO patients using an ocular surface interferometer. In addition, we intended to identify important blink characteristics in NDO patients and assess how effectively subjective symptoms and objective indicators in NDO patients are reflected by indicators.

## 2. Materials and Methods

### 2.1. Participants

From July 2019 to January 2022, a retrospective study was performed on 34 patients (48 cases) who had lacrimal passage intubation (LPI), as well as 24 age- and gender-matched control groups (48 cases). To rule out other eyelid blinking effects, patients with a history of eyelid surgery, ocular disorders, and treatment were removed from the study. Although the relationship between dry eye disease symptoms and signs is weak and variable [7], individuals with dry eye disease were excluded. Age affects blinking [8]; thus, a retrospective study was conducted on 24 control groups (48 cases) with patients of similar ages and genders, with a mean age of 59.5 years in patients in their 40s and 60s who visited clinics for early cataract screening during medical visits.

### 2.2. Quality of Life in Epiphora Patients (E-QOL) Questionnaire

This is an E-QOL questionnaire utilized in this study that focuses on a patient’s symptoms in addition to the Munk scale for patients who are currently visiting the outpatient clinic for epiphora. We identified subjective indicators that are more specific with regard to limitations on everyday living, static activities, and dynamic activities.

The total E-QOL questionnaire score was 60. The degree to which epiphora interferes with daily living was rated out of 10 points, whilst the degree to which it interferes with activities was rated out of 5 points. The daily activity limitation questionnaire has a score of 20 and consists of four questions: to what extent does tearing limit your daily activities? (0 = no limitations, 10 = severe limitations) To what extent does epiphora restrict your interpersonal relationships? (0 = no difficulty, 5 = constant difficulty) and how irritated are you owing to your tears? (0 = no trouble whatsoever, 5 = constant trouble). The static activity restriction questionnaire has a score of 25 and consists of five questions: how much difficulty do you have with reading, driving during the day, driving at night, using a computer, and watching television due to tearing? (0 = no trouble whatsoever, 5 = constant trouble). The dynamic activity restriction questionnaire has a score of 15 and consists of three questions: how much difficulty do you have with the following tasks at work, at home, and outside due to tearing?

### 2.3. Image Acquisition Method

Based on the 20 s videos (600 frames) captured using an ocular surface interferometer, the blink indices were analyzed. In the internal program of the ocular surface interferometer (LipiView^®^, TearScience Inc., Morrisville, NC, USA), the thickness of the lipid layer (LLT), total blink (TB, /20 s), and partial blink (PB, /20 s) were recorded. Spectralis anterior-segment OCT scans (AS-OCT, SPECTRALIS^®^, Heidelberg Engineering, GmbH, Heidelberg, Germany) were utilized to quantify tear meniscus height (TMH), punctal reserve (PR), and punctal diameter.

We define the blink cycle as the blink time (BT), which is the duration of the upper eyelid closing action, and the interblink time (IBT), which is the duration of the upper eyelid remaining open. BT was defined as the sum of the lid closing time (LCT—time taken by the interpalpebral fissure (IPF) to reach the maximum closure from the minimum closure), lid opening time (LOT—time taken by the upper eyelid to change from the minimum to maximum IPF), and closure time (CT—time that the upper eyelid remained completely closed).

**Figure 1:**
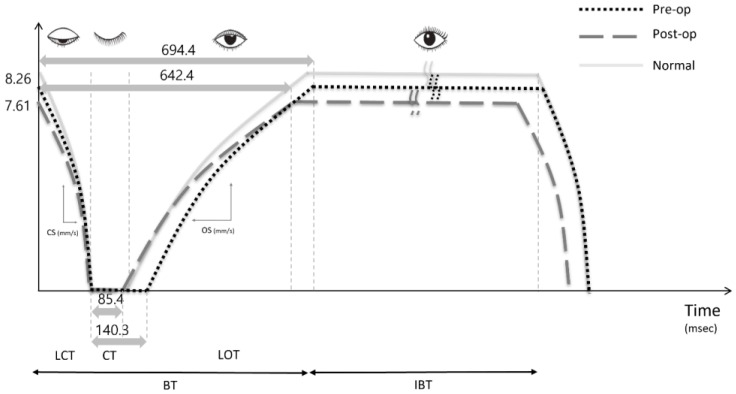
Blink indices graph of before and after lacrimal passage intubation (LPI) obtained using ocular surface interferometer (LipiView^®^). (BT, blink time; LCT, lid closing time; CT, closure time; LOT, lid opening time; IBT, interblink time; IPF, interpalpebral fissure; CS, closing speed; OS, opening speed).

Using these data, the blink index curve could be derived. For both the LCT and LOT periods, at least two time points were selected and calculated to determine the blink dynamic curve’s curvature. Closing speed (CS) and opening speed (OS) were defined as the dynamic index of an eyelid’s closure and opening, respectively. The CS and OS were computed using IPF per LCT (mm/s) and IPF per LOT (mm/s), respectively. The measurements were taken on all blinks that occurred within 20 s, and the results were averaged. The adjusted IPF versus time graph was used to generate each blink curve (Figure 1). For data gathering and analysis, a desktop computer running Windows 11 and video applications were utilized. Every measurement was taken by a single examiner (Y.K.).

The subjective satisfaction of patients following LPI whose TMH was less than 300 μm and who passed irrigation test were deemed successful.

### 2.4. Surgical Technique

As previously described, a single surgeon (H.L.) performed all of the procedures [9]. Using dacryoen doscopy and the insertion of a silicone tube, surgical treatment was performed under general or local anesthesia. After extending the punctum with the punctum dilator and spring scissors and inserting the 0.9 mm diameter probe tip and bent type dacryoendoscope (RUIDO Fiberscope, FiberTech Co., Tokyo, Japan) through the punctum, the internal conditions of the lacrimal duct system were evaluated by passing saline through the upper and lower canaliculus, lacrimal sac, lacrimal duct, and inferior meatus. The obstructive lesion was dislodged by a sheath guided by endoscopy and perfusion solution pressure with a syringe attached to a probe. Under visual guidance, a 0.94 mm-diameter bicanalicular silicone tube (Yoowon Meditec, Seoul, Republic of Korea) was inserted into the sheath. The sheath and tube were retrieved, and both ends of the tube were locked and fixed near the inferior meatus. The tube was removed six months later. According to the clinical course after surgery, levofloxacin 0.5% (cravit^®^; Santen, Osaka, Japan) and fluorometholone 0.1% (Flumetholone^®^; Santen, Osaka, Japan) were prescribed.

### 2.5. Statistical Analysis

SPSS for Windows, version 27.0, was used for all statistical analyses (IBM Corp., Armonk, NY, USA). Parameters were compared using the paired *t*-test, Mann–Whitney test, Kruskal–Wallis test, and Chi-square test. A *p*-value of less than 0.05 was considered statistically significant. Using the Chi-square test, we reported dichotomous outcomes as odds ratios (ORs) and continuous outcomes as the mean and their respective 95% confidence intervals (CIs). Using correlation analysis, the relationships between the categorical variables were examined.

## 3. Results

There were more women than men in the study (male:female = 8:26). The mean age was 59.50 ± 9.53 years, and the direction of occurrence was comparable. The mean Munk scale was 4.04 ± 1.35, the mean Schirmer test value was 34.77 ± 4.62 mm, and the mean BT was 7.74 ± 2.80 s. The average score on the E-QOL questionnaire was 34.81 ± 17.09 out of 60, the score for daily activity restriction was 13.67 ± 4.50, the score for static activity limit was 13.65 ± 7.58, and the score for dynamic activity limit was 8.92 ± 4.50 (Table 1).

Comparable to normal, individuals with NDO had a longer CT, and in the case of unilateral NDO, they had a CT that was approximately 60% longer than the opposite eye. After LPI, this reduced similarly to the usual situation. Comparing CT measurements before and after LPI reveals that it reduced by around 60%, leading to an overall decline in BT. After LPI, the TMH had fallen by more than half in all groups. The preoperative CT was longer in the experimental group than in the control group (89.4 ± 20.0 msec versus 140.3 ± 92.0 msec, *p* = 0.001), but there were no other differences. After surgery, the BT (694.4 ± 135.5 msec, 642.4 ± 116.0 msec, *p* = 0.032) and the CT decreased (140.3 ± 92.0 msec, 85.4 ± 22.7 msec, *p* < 0.001), the TMH decreased significantly from 451.5 ± 254.3 μm to 213.7 ± 112.3 μm, the tear film lipid layer thickness remained unchanged, and the BT, CT, CS and OS values were similar to that of the control group (Table 2).

According to the graph of blink dynamics prior to and after surgery, the IPF along the Y-axis fell to 7.61 mm from 8.26 mm. CT reduced from 140.3 msec to 85.4 msec, which is near normal, and BT decreased from 694.4 msec to 642.4 msec, which is also close to normal (Figure 1).

**Figure 2:**
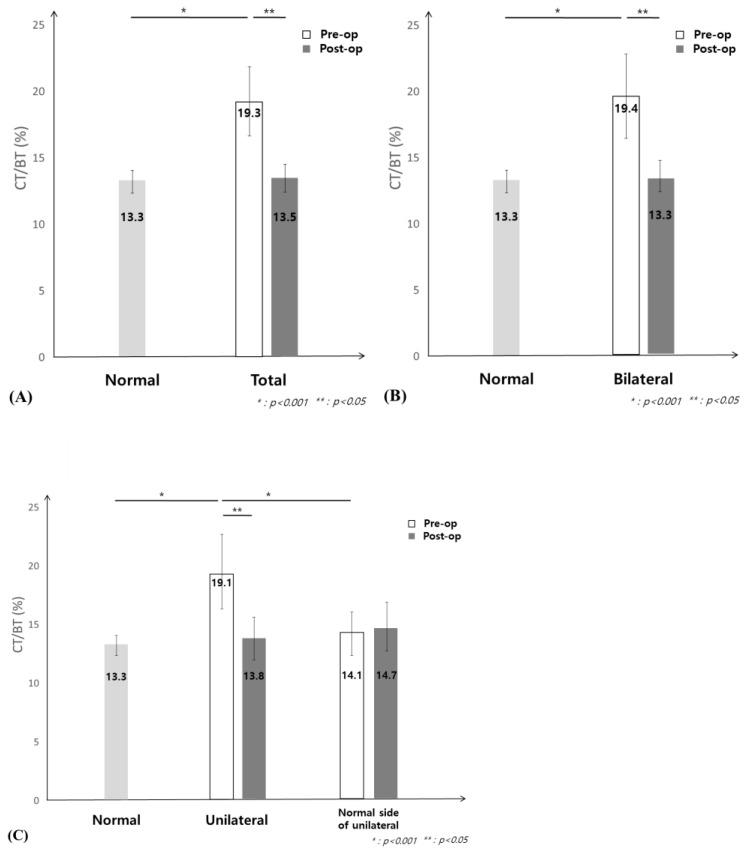
The ratio of index CT during BT (CT/BT) (%) in nasolacrimal duct obstruction (NDO) patients between (**A**) Normal and total NDO patients (**B**) Normal and bilateral NDO patients (**C**) Normal and unilateral NDO patients and the normal side of unilateral NDO patients.

The normal control group had a CT during BT ratio of 13.3%, while patients with NDO had a ratio of 19%. After surgery, the CT during BT ratio returned to normal levels (Figure 2A). Before and after surgery, both unilateral and bilateral patients had the same difference, and it can be seen that they have returned to normal after surgery; therefore, the CT during BT is significant (Figure 2B,C). Interestingly, as reported by Su et al. in the 2018 dry eye blink research, CT was also extended in dry eye disease [10]. Secondary epiphora due to dry eye disease similarly lengthened the CT, but when assessed by the ratio in the blink time, it was 7.3% in dry eye patients and 19.1% in patients with NDO, allowing the distinction between the two groups.

**Figure 3:**
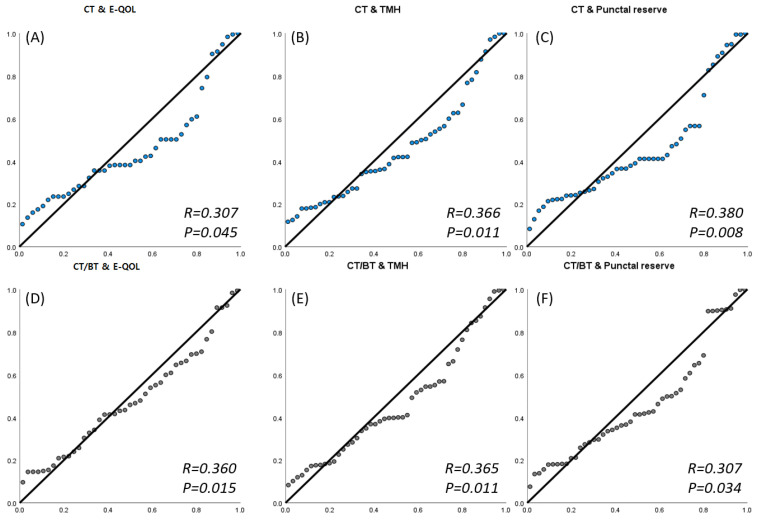
Correlation analysis of the blink index, CT and CT during BT (CT/BT), Quality of Life in Epiphora patients (E-QOL) questionnaire score, tear meniscus height (TMH) and punctal reserve. There are positive correlations with CT and (**A**) Quality of Life in Epiphora patients (E-QOL) questionnaire score, (**B**) tear meniscus height (TMH) and (**C**) punctal reserve. CT during BT (CT/BT) have also positive correlations with CT and (**D**) Quality of Life in Epiphora patients (E-QOL) questionnaire score, (**E**) tear meniscus height (TMH) and (**F**) punctal reserve.

Via an ocular surface-reflecting blink movement study, we sought to identify new indicators that can simultaneously reflect subjective and objective indicators. The blink parameters associated with subjective indicators were BT, CT, and CT during BT; the longer BT and CT were, the greater the ratio of CT during BT, and the stronger the correlation between the E-QOL questionnaire and the objective blink parameters. Objective criteria TMH and punctual reserve showed a positive correlation with the E-QOL questionnaire (Figure 3).

## 4. Discussion

Several attempts have been undertaken to objectively quantify the quantity of tears shed by individuals who complain of various tear symptoms. TMH, punctal diameter and PR are used to objectively measure the number of stagnant tears [1] as well as the irrigation test, dacryocystography, and dacryoendoscopy, which are used to figure out the precise etiology of NDO. A blink accurately reflects the ocular surface and is associated with the stability of the tear film and the health of the ocular surface [4]. Tse et al. tried to evaluate the clinical–anatomical assessment of NDO patients, including blink dynamics. They suggest the BLICK mnemonic as a useful adjunct to the workup of epiphora (Blink dynamics, Lid malposition, Imbrication, Conjunctivochalasis, and Kissing puncta) [11]. Here, we mainly focus on eyelid blinking in NDO patients because it is believed that the discomfort of retained tears has an effect on eyelid blinking.

The authors’ blink profile analysis revealed that NDO patients had a much longer CT and CT during BT than normal controls, and even if only one eye had NDO, the other eye’s indices were significantly longer, too. In other words, the eyes were closed for an extended period of time due to greater tear retention caused by NDO, and it returned to normal after LPI. The TMH had decreased after LPI, and the patient’s CT and CT during BT normalized after surgery. During eyelid closure or opening, positive/negative pressure spikes are formed throughout the lacrimal duct system, and tears flow via the canaliculus to the lacrimal sac and lacrimal duct, according to Sato et al. [12]. It is believed that longer CT and CT during BT in patients with NDO close eyelids more tightly than usual because tears did not drain under the pressure of normal blinking. They have to squeeze their eyelids to drain the tears. Moreover, CT and CT during BT have demonstrated a positive connection with objective markers (TMH, PR) used to evaluate the absolute amount of tearing in NDO patients (Figure 3, *p* < 0.05). Hence, CT and CT during BT are new measures that can establish the severity of tear retention in patients with NDO.

After LPI, patients’ subjective satisfaction may have increased as a result of higher contrast sensitivity, improved vision, decreased blink frequency, and enhanced optical quality [13,14]. Decreased IPF was noted after surgery alongside CT and CT during BT. The exact explanation of this is uncertain; however, it is assumed that the eyes open less due to relative dryness when the LPI-induced tear retention resolves.

For a long time, Munk’s scale has been used to measure the level of subjective discomfort. Unfortunately, the Munk scale now in use only represents the number of tears wiped away on a scale of 0 to 5 points, making it difficult to depict the level of discomfort caused by tears in the patient’s everyday life. More recently, the TEARS scale and WEQOL questionnaire [2,3] have been developed. However, in clinical settings, there are a surprising variety of cases in which the objective quantity of stagnant tears (e.g., TMH) and the patient’s subjective tear-induced symptoms (e.g., subjective satisfaction, decreased vision) are unrelated. For more precise diagnosis, evaluation and proper treatment of NDO patients, parameters that might reflect subjective symptoms and objectively measured tear amount are becoming necessary. Additionally, the WEQOL questionnaire screened for irritated skin due to tears and primarily focused on unpleasant emotions [3]. On the other hand, there is a distinction in that the E-QOL questionnaire distinguished between static and dynamic activities when confirming the discomfort caused by tears during diverse activities with better specificity. The E-QOL questionnaire also showed a positive correlation with objective parameters, TMH and punctual reserve. Additionally, it also has strong correlation with the other objective parameter, CT, CT during BT (Figure 3, *p* < 0.05).

In this study, 33.3% of patients complained of subjective discomfort due to tears, despite their TMH being below 300 μm. Their CT, CT during BT were higher (175.00 msec) than in the normal group (89.4 msec). In addition, among patients with no subjective discomfort, 12.5% of those show a TMH of 300 μm or above. There might be a considerable gap between objective indicators and subjective symptoms of tears, and it is true that there are complicated interactions among the multiple elements. Since TMH is only the summation of tear secretion and excretion, patients suffering from both dry eye disease and NDO could not demonstrate high TMH. Dry eye syndrome is a typical example of a disorder that causes a significant amount of tear evaporation. NDO, combined with this condition, may suggest normal TMH, which may confound the combined NDO for lower TMH than expected in NDO patients with normal tear secretion. This is the reason why we investigate to discover the clinical parameters of subjective discomfort (E-QOL questionnaire) in accordance with objective parameters (TMH, CT, CT during BT).

Therefore, considering that CT and CT during BT demonstrated a favorable connection with the E-QOL questionnaire scores, CT and CT during BT could be considered as a complementary tool to TMH or the E- QOL questionnaire scores of epiphora patients in diagnosis based on this study. Among them, this was strongly associated with dynamic activities, particularly subjective discomfort, and the correlation between the index value and the CT during BT value showing that patients had poorer quality of life due to tears was significant (*p* = 0.001, R = 0.489). Unexpectedly, there was no correlation between Munk’s scale and the blink index and E-QOL questionnaire score measured by the investigators. Considering that CT and CT during BT have a correlation with subjective symptoms, it is expected that further studies comparing Munk’s scale and blink index with the E-QOL questionnaire will be conducted on a larger number of patients. In addition, CT and CT during BT were correlated with TMH and PR but not with the irrigation test, which could indicate the severity of epiphora symptoms. Future applications may include analyzing instantaneous acceleration and electrode signals during blinking and analyzing them in greater detail than 20 s and 600 frames using a method of tracking eyelid blinking by utilizing continuous blink tracking methods (e.g., a wearable blink tracking device) [4].

CT also demonstrated a protracted pattern of secondary tears resulting from dry eye disease; however, CT during BT was distinct from 19.3% in NDO patients and 7.3% in dry eye patients. According to Su et al., patients with dry eye had longer LCT, LOT, CT, PB, and IBT. Hence, patients with dry eyes blink frequently and slowly. In contrast, LCT, LOT, PB, and IBT did not differ significantly between NDO patients. Hence, it may be noticed that NDO patients close their eyes longer than those with dry eyes, but other blinking parameters remain the same. This allows us to differentiate this from CT that has been prolonged by secondary tears. Furthermore, additional studies with a larger sample size are anticipated to distinguish between NDO and dry eyes, as well as tears caused by eyelid malposition and tears caused by external stimuli. In addition to blepharospasm, facial palsy, and NDO, it is anticipated that 20 s of blinking image analysis will substantiate the blink pattern of other ophthalmic diseases.

## 5. Conclusions

CT and CT during BT, which are objective parameters that can also be associated with patients’ subjective epiphora symptoms, are considered to be useful parameters for evaluating NDO patients in clinical settings.

## Figures and Tables

**Table 1:** Demographics and clinical questionnaires of nasolacrimal duct obstruction (NDO) patients.

	Total (*n* = 34, 48 eyes)
Sex (M:F)	8:26
Age (year)	59.50 ± 9.53
Site (OD:OS)	21:27
Unilateral:Bilateral (n)	20:14
Munk’s score	4.04 ± 1.35
Schirmer (mm)	34.77 ± 4.62
BUT (s)	7.74 ± 2.90
E-QOL (60)	34.81 ± 17.09
Daily life restriction (20)	13.67 ± 4.50
Static activity (25)	13.65 ± 7.58
Dynamic activity (15)	8.92 ± 4.65

**Table 2:** Blink pattern and indices of nasolacrimal duct obstruction (NDO) patients before and after lacrimal passage intubation (LPI).

+	Normal (48 eyes)	NDO Patients									P	
		Unilateral (*n* = 20)			Bilateral (*n* = 14)			Total (*n* = 34)				
		Pre	Post	P	Pre	Post	P	Pre	Post	P	Pre	Post
Blink pattern												
TB (/20 s)	8.80 ± 5.20	8.32 ± 5.35	9.82 ± 3.55	0.291	8.32 ± 5.35	9.82 ± 3.55	0.252	7.90 ± 4.80	8.29 ± 3.61	0.601	0.224	0.232
PB (/20 s)	4.12 ± 5.62	4.14 ± 3.66	4.25 ± 3.40	0.482	4.14 ± 3.66	4.25 ± 3.40	0.356	3.85 ± 3.96	4.15 ± 3.43	0.561	0.226	0.446
Blink index												
BT (msec)	679.5 ± 98.6	698.3 ± 125.9	650.0 ± 113.2	0.142	691.7 ± 144.2	636.9 ± 119.8	0.086	694.4 ± 135.5	642.4 ± 116.0	0.022	0.715	0.103
LCT (msec)	134.8 ± 35.2	128.3 ± 34.7	125.0 ± 38.9	0.725	136.9 ± 50.0	129.8 ± 26.2	0.433	133.3 ± 44.0	127.8 ± 31.8	0.394	0.746	0.314
CT (msec)	89.4 ± 20.0	140.0 ± 97.1	86.7 ± 16.8	**0.017**	140.5 ± 90.0	84.5 ± 26.4	**0.001**	140.3 ± 92.0	85.4 ± 22.7	**<0.001**	**0.001**	0.377
LOT (msec)	455.3 ± 81.9	430.0 ± 86.5	438.3 ± 99.9	0.770	414.3 ± 85.3	422.6 ± 105.8	0.696	420.8 ± 85.2	429.2 ± 102.6	0.662	0.053	0.183
IBT (msec)	14,563.6 ± 789.0	14,835.0 ± 2772.9	15,993.3 ± 1599.9	0.129	14,164.3 ± 4295.1	13,592.9 ± 3244.0	0.437	14,459.7 ± 3729.7	14,593.1 ± 2917.3	0.779	0.190	0.947
CS (mm/s)	68.54 ± 18.92	69.45 ± 15.90	68.68 ± 24.15	0.873	63.84 ± 17.46	58.96 ± 15.72	0.137	66.18 ± 16.89	63.01 ± 20.03	0.246	0.737	0.178
OS (mm/s)	19.65 ± 3.93	20.39 ± 4.13	19.08 ± 5.81	0.310	20.65 ± 6.58	18.58 ± 5.83	0.060	20.54 ± 5.64	18.79 ± 5.77	0.117	0.511	0.410
IPF (mm)	8.74 ± 1.33	8.51 ± 1.16	7.92 ± 1.17	**0.022**	8.09 ± 1.26	7.39 ± 1.43	**0.002**	8.26 ± 1.23	7.61 ± 1.34	**0.015**	0.052	**0.001**
CT/BT (%)	13.16	20.05	13.34		20.31	13.27		20.20	13.29			
TMH (μm)	-	500.25 ± 232.93	202.20 ± 94.38	**<0.001**	416.61 ± 267.22	221.89 ± 124.60	**<0.001**	451.46 ± 254.34	213.69 ± 112.32	**<0.001**	-	-

NDO, nasolacrimal duct obstruction; TB, total blink; PB, partial blink; BT, blink time; LCT, lid closing time; CT, closure time; LOT, lid opening time; IBT, interblink time; IPF, interpalpebral fissure; CS, closing speed; OS, opening speed. Bold: *p* < 0.05.

## Data Availability

The data presented in this study are available upon request from the corresponding author. The data are not publicly available due to privacy.
